# Correlating Global Gene Regulation to Angiogenesis in the Developing Chick Extra-Embryonic Vascular System

**DOI:** 10.1371/journal.pone.0007856

**Published:** 2009-11-17

**Authors:** Sophie Javerzat, Mélanie Franco, John Herbert, Natalia Platonova, Anne-Lise Peille, Véronique Pantesco, John De Vos, Said Assou, Roy Bicknell, Andreas Bikfalvi, Martin Hagedorn

**Affiliations:** 1 INSERM U920, Laboratoire des Mécanismes Moléculaires de l'Angiogenèse, Université Bordeaux 1, Talence, France; 2 Université Bordeaux 1, Talence, France; 3 Institut de Recherche en Biothérapie, Hôpital Saint-Eloi, CHU de Montpellier, Montpellier, France; 4 Molecular Angiogenesis Group, Institute of Biomedical Research, University of Birmingham, Medical School, Birmingham, United Kingdom; Ecole Normale Supérieure de Lyon, France

## Abstract

**Background:**

Formation of blood vessels requires the concerted regulation of an unknown number of genes in a spatial-, time- and dosage-dependent manner. Determining genes, which drive vascular maturation is crucial for the identification of new therapeutic targets against pathological angiogenesis.

**Methology/Principal Findings:**

We accessed global gene regulation throughout maturation of the chick chorio-allantoic membrane (CAM), a highly vascularized tissue, using pan genomic microarrays. Seven percent of analyzed genes showed a significant change in expression (>2-fold, FDR<5%) with a peak occurring from E7 to E10, when key morphogenetic and angiogenic genes such as BMP4, SMO, HOXA3, EPAS1 and FGFR2 were upregulated, reflecting the state of an activated endothelium. At later stages, a general decrease in gene expression occurs, including genes encoding mitotic factors or angiogenic mediators such as CYR61, EPAS1, MDK and MYC. We identified putative human orthologs for 77% of significantly regulated genes and determined endothelial cell enrichment for 20% of the orthologs *in silico*. Vascular expression of several genes including ENC1, FSTL1, JAM2, LDB2, LIMS1, PARVB, PDE3A, PRCP, PTRF and ST6GAL1 was demonstrated by *in situ* hybridization. Up to 9% of the CAM genes were also overexpressed in human organs with related functions, such as placenta and lung or the thyroid. 21–66% of CAM genes enriched in endothelial cells were deregulated in several human cancer types (*P*<.0001). Interfering with PARVB (encoding parvin, beta) function profoundly changed human endothelial cell shape, motility and tubulogenesis, suggesting an important role of this gene in the angiogenic process.

**Conclusions/Significance:**

Our study underlines the complexity of gene regulation in a highly vascularized organ during development. We identified a restricted number of novel genes enriched in the endothelium of different species and tissues, which may play crucial roles in normal and pathological angiogenesis.

## Introduction

During development or tissue remodeling, growth of blood vessels by sprouting and intussusception is essential for adaptation to increasing needs of oxygen and nutrients. In pathological conditions such as cancer, developmental genes are reactivated in the endothelium, leading to *de novo* growth of blood vessel to supply the tumor with nutrients [1], [2]. Detailed knowledge about differences and similarities of gene regulation during normal and pathological angiogenesis is essential to design new drugs aiming at specific modulation of blood vessel growth and function. Gene expression profiles of vascular endothelial cells (ECs) have been determined after isolation of ECs from specific organs or tumors, based on the expression of known EC-markers or incorporation of fluorescent dyes [3], [4], [5], after stimulation with angiogenesis modulators [6], or after treatment with atorvastatin [7]. Furthermore, microarrays and serial analysis of gene expression (SAGE) have been used to identify genes specific for normal and tumor blood vessels [8], [9], and recently, for freshly isolated endothelial cells and lymphatic endothelial cells [10]. Substractive transcriptomic analysis has recently led to the identification of 58 genes specific for the microvasculature [11]. However, all the above-mentioned studies need experimental manipulation of ECs at some point prior analysis of gene expression (e.g. FACS sorting). Furthermore, gene expression signatures have not yet been associated with a particular form of angiogenesis (eg. sprouting vs. intussusception). During vascular development, cell-to-cell communications take place between ECs, pericytes, vascular smooth muscle cells, epithelial cells or bone marrow-derived cells [12]. These interactions influence gene expression in ECs. Separation of the endothelial compartment from the rest of the organ might compromise EC gene expression patterns and exclude important genes with angiomodulatory activities. Indeed, factors produced by stroma cells in contact with capillaries play important roles in the establishment, maintenance and branching of the vasculature [13], [14], [15], [16], [17], [18]. We took advantage of the CAM as a unique, accessible vascularized organ, whose capillary bed matures in well-defined steps [19], [20], [21], [22], [23], [24] and determined its transcriptome.

We provide evidence for EC-enrichment for a large number of regulated genes and show that the majority of them are deregulated in highly vascularized tumors such as glioblastoma. Our data thus constitute a valuable resource to streamline further research of candidate molecules susceptible to mediate angiogenesis in pathological conditions.

## Results

### Morphological and molecular characterization of CAM development

**Figure 1:**
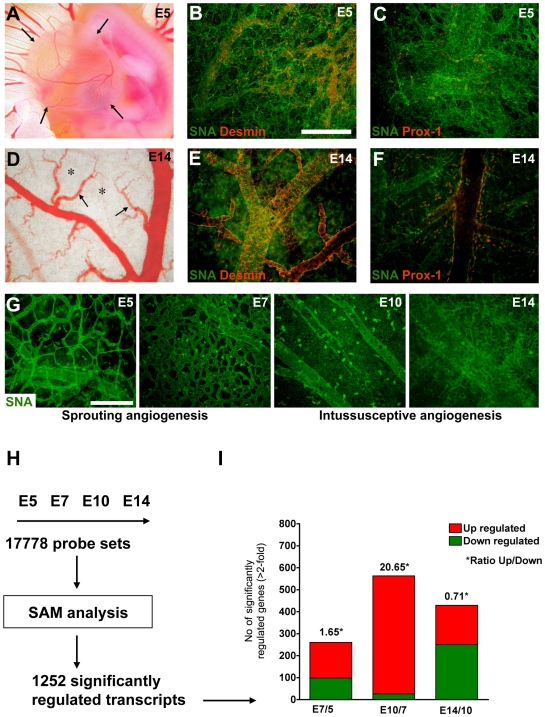
Morphological description of CAM development and gene regulation. A) By day 5 of development, the allantoic vesicle (arrows) has emerged and contains a dense vascular network (biomicroscopy, ×40). B, C) Immunofluorescence of CAM whole-mounts using markers for endothelial cells (SNA lectin), pericytes (desmin) and lymphatic cells (Prox-1). At this stage, the capillary network appears unstructured; few pericytes are associated with the vessels and single lymphatic endothelial cells are scattered throughout the tissue. D) By day 14, the CAM vasculature is hierarchized, a clearly structured vascular tree has developed; feeder vessels (arrows) project into the two-dimensional capillary layer (asterisks; ×63). E, F) Immunofluorescence analysis shows dense pericyte coverage of larger vessels and the existence of an organized lymphatic vascular network. G) Evolution of the capillary network from E5 to E14. Note progressive densification of the capillary plexus from E5 to E7 by means of sprouting angiogenesis; and from E7 to E10 increasingly by intussusceptive angiogenesis [97]. From E10 to E14, little morphological changes are observed in the capillary layer, but some feeder vessels show more ramifications (bars: 200 µm). H) Gene expression analysis during CAM development and Significant Analysis of Microarrays (SAM) identified 1252 probe sets regulated more than 2-fold during the compared periods. I) Ratios of up and downregulated genes reveals a highly active growth phase between E7 and E10 (>20 times genes upregulated), followed by a shift to gene downregulation from E10 to E14.

Adaptation of the CAM vascular bed to increasing oxygen needs of the embryo follows a stereotyped pattern of development. Growth of the initial vascular plexus occurs mainly through sprouting angiogenesis between E5 and E7, followed by a phase of intussusceptive angiogenesis, and then the network expands without further changes in complexity [21], [22], [23]. This is accompanied by increasing endothelial cell proliferation, which peaks around E10 and strongly decreases after E13 [19], [20]. The capillary network of the allantoic vesicle at E5 (hereafter also called CAM) is clearly visible by biomicroscopy (Fig. 1 A). *Sambucus nigra* lectin staining confirms an immature vasculature at E5 with few pericytes and Prox-1 positive cells scattered throughout the tissue (Fig. 1 B, C, G). Pericytes and lymphatic endothelial cells associate with vascular structures during the following days, and an organized vascular tree is established by day 14 (Fig. 1 D–F). Density and ramification of the vascular plexus increase constantly throughout CAM development (Fig. 1 G).

We compared global gene expression changes of the CAM between E5, E7, E10 and E14 using the recently released Affymetrix chicken GeneChips.

Significance Analysis of Microarrays (SAM) analysis identified 1252 probe sets which detect significant gene expression changes throughout CAM development (>2-fold, FDR<5%). This corresponds to 7% of the original filtered input data set (17778 probe sets). Two hundred and sixty probes represented genes regulated between E5 and E7 (162 up, 98 down, ratio up/down: 1.65-fold). The period from E7 to E10 is a particular highly active phase as reflected by the large number of upregulated genes (537 up, 26 down, ratio up/down: 20.65-fold). Between E10 and E14, the ratio was inversed with 179 transcripts upregulated and 250 downregulated (ratio 0.71). Selected regulated genes with established or potential roles in angiogenesis between indicated stages are listed in tables 1–3 (for complete list of regulated genes, see Supplementary table S1).

**Table 1:** Selected regulated genes during the early phase of CAM development (E5 to E7).

Gene ID	Fold Change	Gene Symbol	Gene Title
Gga.540.1.S1_at	8,49	MGP	matrix Gla protein
Gga.19409.1.S1_at	6,33	COL4A2	collagen, type IV, alpha 2
Gga.496.1.S1_at	5,00	RELN	reelin
Gga.2587.1.S1_at	4,82	NOV	nephroblastoma overexpressed gene
GgaAffx.25229.1.S1_at	4,56	VWF	von Willebrand factor
GgaAffx.3009.1.S1_s_at	4,01	SEMA6D	Sema domain, transmembrane domain (TM), and cytoplasmic domain, (semaphorin) 6D
Gga.3219.1.S1_at	3,92	FIGF	c-fos induced growth factor (vascular endothelial growth factor D)
Gga.566.1.S1_at	3,74	ITGA1	integrin, alpha 1
Gga.4675.1.S2_at	3,53	SDC2	syndecan 2
Gga.7528.1.S1_at	3,52	KLF2	Kruppel-like factor 2 (lung)
Gga.19409.1.S1_s_at	3,14	COL4A2	collagen, type IV, alpha 2
Gga.1846.1.S2_at	3,12	SEMA3C	sema domain, immunoglobulin domain (Ig), short basic domain, secreted, (semaphorin) 3C
Gga.12186.1.S1_at	3,11	LYVE1	lymphatic vessel endothelial hyaluronan receptor 1
Gga.1761.1.S1_at	2,99	GPR116	G protein-coupled receptor 116
Gga.6311.1.S1_at	2,97	HEY1	hairy/enhancer-of-split related with YRPW motif 1
Gga.2827.2.S1_a_at	2,91	TIMP3	TIMP metallopeptidase inhibitor 3 (Sorsby fundus dystrophy, pseudoinflammatory)
Gga.7231.1.S1_at	2,42	SOX17	SRY (sex determining region Y)-box 17
GgaAffx.24123.1.S1_at	2,40	PDE3A	phosphodiesterase 3A, cGMP-inhibited
Gga.679.1.S1_at	2,38	VLDLR	very low density lipoprotein receptor
GgaAffx.25881.1.S1_at	2,34	PECAM1	platelet/endothelial cell adhesion molecule (CD31 antigen)
Gga.5541.1.S1_at	2,18	PECAM1	platelet/endothelial cell adhesion molecule
Gga.15805.1.S1_at	2,08	FLT4	fms-related tyrosine kinase 4
Gga.12104.1.S1_at	2,03	RAMP2	receptor (G protein-coupled) activity modifying protein 2
*Gga.19305.1.S1_at*	*0,12*	*RSPO3*	*R-spondin 3 homolog (Xenopus laevis)*
*Gga.4830.1.S1_at*	*0,37*	*SFRP1*	*secreted frizzled-related protein 1*
*Gga.5170.1.S1_s_at*	*0,42*	*PODXL*	*podocalyxin-like*

A selection of relevant genes during the early period of CAM development are shown (for all significantly regulated genes, see Supplementary table S1). Downregulated genes are in italic.

**Table 2 pone-0007856-t002:** Selected regulated genes during the intermediate phase of CAM development (E7 to E10).

Gene ID	Fold Change	Gene Symbol	Gene Title
Gga.686.1.S1_at	8,40	BMP4	bone morphogenetic protein 4
GgaAffx.20323.1.S1_at	7,32	DAG1	dystroglycan
Gga.8140.1.S1_at	4,37	FGFBP1	fibroblast growth factor binding protein 1
Gga.1964.1.S1_at	3,55	MYO10	myosin X
Gga.4048.1.S1_at	3,49	ID3	inhibitor of DNA binding 3, dominant negative helix-loop-helix protein
Gga.2875.2.S1_a_at	3,34	ACVR1	activin A receptor, type I
Gga.889.1.S1_at	3,12	ITGA3	integrin, alpha 3 (antigen CD49C, alpha 3 subunit of VLA-3 receptor)
Gga.18461.1.S1_s_at	2,76	PTPRF	protein tyrosine phosphatase, receptor type, F
Gga.4082.1.S1_at	2,67	SMO	smoothened homolog (Drosophila)
Gga.496.1.S1_at	2,59	RELN	reelin
Gga.11518.1.S1_at	2,58	—	—
Gga.3381.1.S2_at	2,58	HOXA3	homeobox A3
Gga.1030.1.S1_at	2,57	MYC	v-myc myelocytomatosis viral oncogene homolog (avian)
Gga.3673.1.S2_at	2,49	LDB2	LIM domain binding 2
Gga.3035.1.S1_at	2,41	EPAS1	endothelial PAS domain protein 1
GgaAffx.24545.1.S1_at	2,33	ENC1	ectodermal-neural cortex (with BTB-like domain)
GgaAffx.20877.1.S1_at	2,29	SOX7	SRY (sex determining region Y)-box 7
Gga.977.1.S1_at	2,25	PTRF	polymerase I and transcript release factor
Gga.19289.1.S1_at	2,07	PRCP	Prolylcarboxypeptidase (angiotensinase C)
GgaAffx.20978.1.S1_s_at	2,05	FGFR2	fibroblast growth factor receptor 2
Gga.9700.1.S1_at	2,05	TSPAN7	tetraspanin 7
*Gga.19409.1.S1_at*	*0,01*	*COL4A2*	*collagen, type IV, alpha 2*
*Gga.3219.1.S1_at*	*0,23*	*FIGF*	*c-fos induced growth factor (vascular endothelial growth factor D)*

A selection of relevant genes during the intermediate period of CAM development are shown (for all significantly regulated genes, see Supplementary table S1). Downregulated genes are in italic.

**Table 3:** Selected regulated genes during the late phase of CAM development (E10 to E14).

Gene ID	Fold Change	Gene Symbol	Gene Title
Gga.19409.1.S1_at	89,38	COL4A2	collagen, type IV, alpha 2
Gga.3219.1.S1_at	7,42	FIGF	c-fos induced growth factor (vascular endothelial growth factor D)
Gga.5334.1.S1_a_at	2,66	RGS4	regulator of G-protein signaling 4
GgaAffx.12606.1.S1_s_at	2,56	JAM2	junctional adhesion molecule 2
GgaAffx.22422.1.S1_s_at	2,20	AMOT	angiomotin
Gga.977.1.S1_at	2,09	PTRF	polymerase I and transcript release factor
*Gga.16689.1.S1_s_at*	*0,18*	*PARVB*	*parvin, beta*
*Gga.805.2.S1_a_at*	*0,30*	*EPHA3*	*EPH receptor A3*
*Gga.1964.1.S1_at*	*0,30*	*MYO10*	*myosin X*
*Gga.5002.1.S1_at*	*0,32*	*MDK*	*midkine (neurite growth-promoting factor 2)*
*GgaAffx.20323.1.S1_at*	*0,33*	*DAG1*	*dystroglycan*
*GgaAffx.1193.1.S1_s_at*	*0,33*	*LAMA1*	*laminin, alpha 1*
*Gga.3035.1.S1_at*	*0,38*	*EPAS1*	*endothelial PAS domain protein 1*
*Gga.1846.1.S1_at*	*0,42*	*SEMA3C*	*sema domain, immunoglobulin domain (Ig), short basic domain, secreted, (semaphorin) 3C*
*Gga.18504.1.S1_at*	*0,43*	*PARVA*	*parvin, alpha*
*Gga.3973.1.S1_at*	*0,44*	*TWIST1*	*twist homolog 1 (Drosophila)*
*Gga.5109.1.S1_s_at*	*0,46*	*MYCN*	*v-myc myelocytomatosis viral related oncogene, neuroblastoma derived (avian)*
*Gga.2200.1.S1_at*	*0,49*	*CYR61*	*cysteine-rich, angiogenic inducer, 61*
*Gga.19934.1.S1_at*	*0,49*	*SNAI2*	*snail homolog 2 (Drosophila)*
*Gga.1846.1.S2_at*	*0,49*	*SEMA3C*	*sema domain, immunoglobulin domain (Ig), short basic domain, secreted, (semaphorin) 3C*
*Gga.2679.2.S1_a_at*	*0,49*	*FBLN1*	*fibulin 1*
*GgaAffx.21814.1.S1_s_at*	*0,50*	*LIMS1 /// LOC771176*	*LIM and senescent cell antigen-like domains 1 /// hypothetical protein LOC771176*

A selection of relevant genes during the late period of CAM development are shown (for all significantly regulated genes, see Supplementary table S1). Downregulated genes are in italic.

### Early growth phase: E5 to E7

The early phase of the CAM is characterized by extensive growth of the vascular network, essentially by means of sprouting angiogenesis. Important genes controlling morphogenesis and blood vessel formation in vertebrates were found upregulated. These include critical transcription factors such as SOX17, HEY1, KLF2, members of the semaphorin family like SEMA6D, SEMA3C, and genes encoding for various proteins required for endothelial cell function such as RAMP2, NOV, VWF, PECAM1 and MGP (Table 1). At the same time, the establishment of the CAM lymphatic vascular system is reflected by the concerted activation of major factors which positively drive lymphangiogenesis such as RELN, its receptor VLDLR, LYVE1 and FIGF and its receptor FLT4. Interestingly, COL4A2, the gene encoding the precursor of the endogenous angiogenesis inhibitor canstatin [25], was also upregulated during this period. Other essential angiogenic modulators such as RSPO3 and SFRP1 were negatively regulated between E5 and E7.

**Figure 2:**
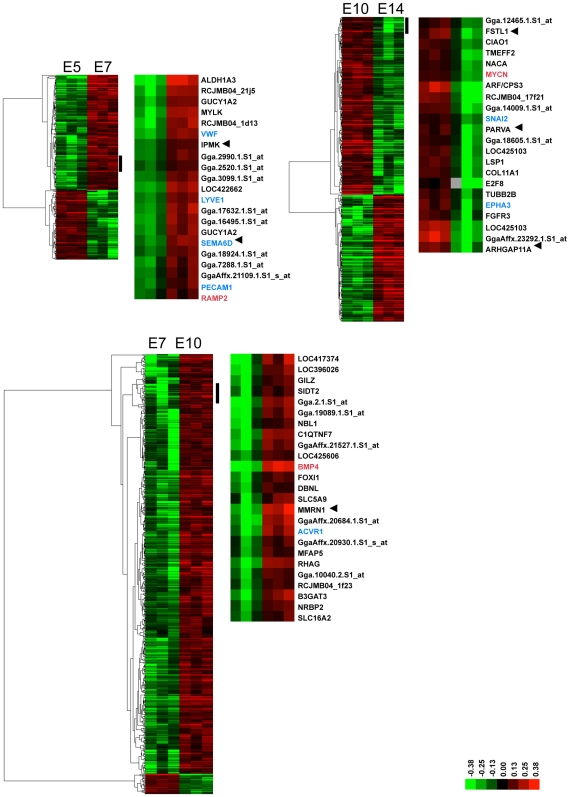
Co-expression of established and potential regulators of angiogenesis during CAM maturation. Cluster analysis of expression values of all probe sets representing significantly regulated genes between indicated periods. For each indicated period, the left cluster shows all probe sets, the right shows the transcripts of the node indicated by a black bar. The nodes correspond to transcripts co-expressed with genes known to control vascular morphogenesis (gene symbol in red). In blue are co-expressed genes with established function in the same process (e.g. lymphangiogenesis: RAMP2 and LYVE1), which physically interact (BMP4 and ACVR1) or which participate in the same signaling chain (MYCN, SNAI2). Arrows indicate genes enriched in endothelial cells (data from Supplementary table S3).

Co-expression/regulation of angiogenic genes was further evidenced on the signal level by a cluster analysis of significantly regulated genes (Fig. 2). Numerous genes are co-expressed with genes with established roles in angiogenesis (shown in red). Between E5 and E7, we found the positive angiogenic and lymphangiogenic regulator RAMP2 [26], [27] co-regulated with LYVE1, PECAM1, VWF, SEMA6D and genes, which have not yet been described to play a role in blood vessel growth such as IPMK (inositol polyphosphate multikinase).

To obtain general information about biological processes enriched or activated between E5 and E7, we submitted all genes regulated during this to the DAVID database. DAVID associates Gene Ontology terms with a set of genes and calculates enrichment compared to the rest of the genome. Cell adhesion was found to be the predominant process, which associates 8 critical genes including ITGA1, ITGA6, RELN and LYVE1 (extracellular link domain containing 1) (Supplementary figure S2). Cell-matrix interactions are of fundamental importance during vascular development (for review see [28], [29]). These results suggest that the early developing CAM is especially active in regard to matrix remodeling and adhesion.

### Intermediate (E7 to E10) and late growth phase (E10 to E14)

Between E7 and E10, the CAM vasculature undergoes active remodeling associated with a high index of EC proliferation [19], [20]. This is reflected by the fact that very few genes were downregulated during that period (Table 2 and Supplementary table S1).

EPAS1 (hypoxia-inducible factor 2α) was significantly regulated throughout the development and peaked around E10: 2.41-fold up from E7 to E10 (qPCR: 2.65-fold), 0.38-fold down from E10 to E14 (qPCR: 0.18-fold). Another essential angiogenesis modulator, CYR61, declined together with EPAS1 from E10 to E14: 0.49-fold (qPCR: 0.51-fold).

Interestingly, FIGF (encoding vascular endothelial growth factor D) showed two peaks of regulation (3.92-fold up from E5 to E7, down 0.23-fold from E7 to E10 and up 7.42-fold again from E10 to E14; Table 3). The most strongly downregulated gene (0.01-fold) during that period was COL4A2, the gene encoding the precursor for the angiogenesis inhibitor canstatin.

BMP4 was one of the highest upregulated genes between E7 and E10 and co-regulates with the gene encoding its high affinity receptor, ACVR1 (activin A receptor, type I). In this cluster was also an EC-expressed gene, MMRN1 (multimerin 1), which encodes a large protein with a similar structure to von Willebrand factor [30].

Interestingly, the Gene Ontology analysis found enrichment of genes with known roles in morphogenesis and vascular development (1.99-times) amongst the genes upregulated during the period from E7 to E10. Again, this includes BMP4 and ACVR1, together with SMO, HOXA3 and other morphogens (Supplementary figure S2). This is likely correlated with an increasing complexity of the CAM vasculature associated with increasing differentiation of specific cell populations. Between E10 and E14, a reduction of MYCN is observed, together with its target gene SNAI2. Not present in the Cluster, but also downregulated during this period was TWIST1 (0.46-fold). These three genes are functionally linked and are essential for normal blood (and lymphatic) vessel formation in *Xenopus laevis* [31]. Gene ontology analysis performed on the genes downregulated from E10 to E14, identified “mitosis” as the most enriched biological process. Numerous genes essential for coordinated cell proliferation including CCNA2, CCNB2 and MAD2 are downregulated during this phase, confirming that the CAM has entered a more quiescent developmental stage.

### Ortholog identification and *in silico* prediction of EC vs Non-EC expression

To focus on genes, which may have conserved function between vertebrates, we next applied three algorithms to identify human orthologs of chicken genes. All genes significantly regulated at any stage of CAM development were analyzed by an automated ortholog screen. This procedure led to a more precise identification of previously unannotated or poorly annotated chicken genes. At the time of analysis (March 2009), our method assigned a high quality prediction for about 77.55% of the regulated genes (Supplementary table S2).

The CAM is a highly vascularized tissue and mainly contains capillaries and larger vessels. ECs therefore constitute their main cellular component, but epithelial cells and other stromal cells (e.g. unclassified macrophages) are also present. We determined whether any given ortholog regulated during CAM maturation had preferential expression in endothelial cells compared to non-endothelial cells using a recently published *in silico* method, which compares sequence of any given gene to two pools of EST cDNA libraries, endothelial ESTs and non-endothelial ESTs, followed by an FDR-based approach [32]. This analysis assigned EC-enrichment of more than two-fold to 178 unique transcripts. Amongst the most EC-specific genes were 35 transcripts with absolute EC-specificity (e.g. no EST clones isolated from non-endothelial cells have been reported), including JAM2, SOX7, SOX17, HEY1, SEMA6D and UNC5B (Table 4 and Supplementary table S3). Note that genes encoding proteins already used as endothelial markers are identified using this approach and display very high EC-enrichment ratios (VWF: no non-EC clones found; PECAM1: 179-fold enrichment). Accordingly, we found 297 unique transcripts in the Non-EC pool, including MDK, CALR and COL4A2 (Supplementary table S4).

**Table 4:** Endothelial enrichment of putative human orthologs of CAM genes.

Significant regulation period	Gene	Human product	EC-specific expression (q-value)	EC/Non-EC ratio
**Up E10 to E14**	**JAM2**	**junctional adhesion molecule 2**	**0**	**—**
Up E7 to E10	MMRN1	multimerin 1	0	—
**Up E7 to E10**	**SOX7**	**SRY (sex determining region Y)-box 7**	**0**	**—**
Up E5 to E7	VWF	von Willebrand factor	0	—
Up E5 to E7	HEY1	hairy/enhancer-of-split related with YRPW motif 1	0.01	—
Up E5 to E7	C8orf4	chromosome 8 open reading frame 4	0.1	—
Up E10 to E14	GPR137B	G protein-coupled receptor 137B	0.1	—
Up E5 to E7	SOX17	SRY (sex determining region Y)-box 17	0.1	—
Down E7 to E10	CENPL	centromere protein L	0.29	—
Down E10 to E14	DISP1	dispatched homolog 1 (Drosophila)	0.29	—
Down E10 to E14	DTL	denticleless homolog (Drosophila)	0.29	—
Up E7 to E10	MXD1	MAX dimerization protein 1	0.29	—
**Up E5 to E7**	**PDE3A**	**phosphodiesterase 3A, cGMP-inhibited**	**0.29**	**—**
Up E5 to E7	SEMA6D	sema domain, transmembrane domain (TM), and cytoplasmic domain, (semaphorin) 6D	0.29	—
Up E7 to E10	SHE	Src homology 2 domain containing E	0.29	—
Up E7 to E10	UNC5B	unc-5 homolog B (C. elegans)	0.29	—
Down E10 to E14	A2M	alpha-2-macroglobulin	0	208.91
**Up E5 to E7**	**PECAM1**	**platelet/endothelial cell adhesion molecule**	**0**	**179.00**
Up E7 to E10	ARSJ	arylsulfatase family, member J	0.01	23.00
Up E10 to E14	RGS4	regulator of G-protein signaling 4	0	20.23
Up E5 to E7	EMP1	epithelial membrane protein 1	0	19.87
**Up E7 to E10**	**PRCP**	**prolylcarboxypeptidase (angiotensinase C)**	**0**	**17.52**
Up E7 to E10	EFEMP1	EGF-containing fibulin-like extracellular matrix protein 1	0	15.91
Down E5 to E7	TTK	TTK protein kinase	0.12	13.71
**Down E5 to E7**	**PODXL**	**podocalyxin-like**	**0**	**13.14**
Down E10 to E14	WDSOF1	WD repeats and SOF1 domain containing	0.01	12.87
**Up E7 to E10**	**PTRF**	**polymerase I and transcript release factor**	**0**	**12.65**
**Up E7 to E10**	**LDB2**	**LIM domain binding 2**	**0**	**8.68**
Up E5 to E7	ITGA6	integrin, alpha 6	0.01	6.95
**Down E10 to E14**	**PARVB**	**parvin, beta**	**0.05**	**6.66**
Up E7 to E10	MYC	v-myc myelocytomatosis viral oncogene homolog (avian)	0.03	6.08
**Down E10 to E14**	**CYR61**	**cysteine-rich, angiogenic inducer, 61**	**0**	**6.01**
Up E7 to E10	TSPAN7	tetraspanin 7	0.18	5.86

A selection of CAM development genes with high EC-enrichment (>5). Not that numerous genes (including JAM2, SOX7, VWF, PECAM1 and UNC5B) had absolute EC specificity (zero non-EC EST assignments). Genes with validated EC expression by *in situ* hybridization are marked in bold (complete list of EC-enriched genes are in Supplementary table S3).

### *In situ* hybridization

**Figure 3:**
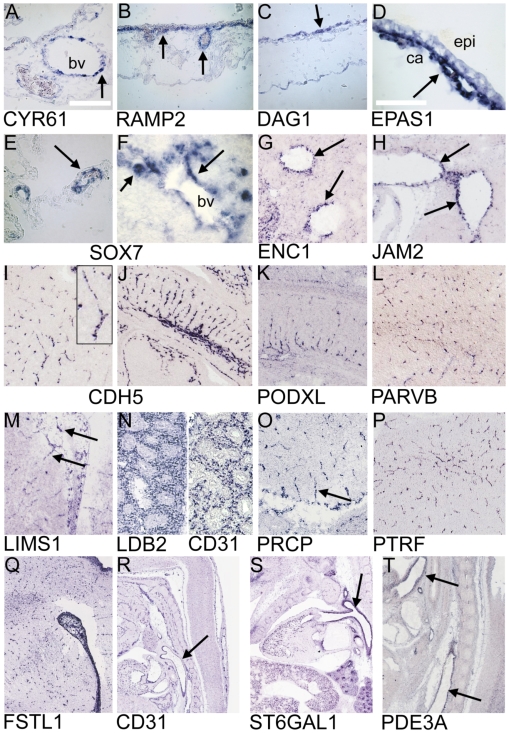
*In situ* hybridization of CAM development genes. A–F) Specific expression of indicated genes in the chick embryo (CAM: A–E; E4 embryo: F). CYR61, RAMP2, EPAS1 and SOX7 are expressed by ECs of the CAM (arrows). C) DAG1 is strongly expressed in epithelial cells adjacent to the capillary network of the CAM (arrow). F) SOX7 transcripts were also found in E4 embryonic vessels (arrows); (epi = Epithelium, ca = capillary network, bv = blood vessel. A–C: scale bar = 100 µm, D–F, scale bar = 50 µm; A+C: E7, B: E10; D: E10). G–T) Blood vessels of different origin positive for indicated transcripts in mouse E14.5 embryos (sagittal sections). G) ENC1 marks liver blood vessels (arrows) and JAM2 labels inferior vena cava (arrows, H). Highly endothelial-specific CDH5 (VE-Cadherin) and PECAM1 (CD31) served as positive controls to demonstrate morphological appearances of micro- and macrovascular structures. Insert in I shows a typical brain capillary at higher magnification. PARVB, LIMS1, PRCP, PTRF, FSTL1 were found in capillaries in brain parenchyma (L, M, O, P, Q), with a pattern comparable to CDH5 (I). LDB2 exhibits a staining similar to CD31 in lung (N), and PODXL probes label tongue capillaries (K). ST6GAL1 and PDE3A strongly mark large blood vessels, such as the aorta (arrows), comparable to the known marker PECAM1 (CD31). All images were taken at digital zoom levels, which best demonstrated vascular patterns (www.genepaint.org).

Endothelial expression of selected genes was further validated by *in situ* hybridization in the developing CAM at different stages (Fig. 3 A–E). Expression of CYR61 was found in CAM ECs of larger conduct vessels, staining of capillaries was less pronounced. This was also confirmed in sections of E4 chicken embryos, where strong CYR61 signal was present in ECs of larger blood vessels (data not shown). RAMP2 and EPAS1 showed intense expression in ECs of the capillary layer and also in feeder vessels. SOX7 transcripts were detected in ECs of E7 CAM and E4 chick embryonic vessels (Fig. 3 F). DAG1 (encoding dystroglycan) was strongly expressed in epithelial cells adjacent to the capillary network, but not in ECs, even though it has been shown that dystroglycan can be produced by ECs and plays distinct roles in angiogenesis [33].

ENC1 marks liver blood vessels (arrows). JAM2 labels aortic trunk (not shown) and inferior vena cava (arrows, H). PARVB, LIMS1, PRCP, PTRF and FSTL1 were found in capillaries in brain parenchyma, with a similar pattern as CDH5. LDB2 exhibits a staining similar to CD31 in lung and PODXL probes label tongue capillaries. ST6GAL1 and PDE3A strongly mark large blood vessels, such as the aorta (arrows).

### Conserved gene expression between CAM and human organs

Transcripts regulated during CAM maturation were compared to sets of genes overexpressed in human tissues. The developing CAM shared the highest number of genes with placenta (9.44%; 1.63-fold over the mean percentage of all organs together), followed by the lung (8.05%, 1.39-fold) and the highly vascularized thyroid (7.42%; 1.28-fold) (Table 5). All other organs had lower ratios, with the skin being the organ with fewest common genes (3.82%). We then determined to which extent EC-enriched genes regulated during CAM development where also enriched in human organs. Here again, placenta contained the highest number of shared genes (28%; 1.54-fold over mean enrichment), followed by the thyroid (26%, 1.44-fold) and the lung (20%; 1.13-fold). Skin and kidney were the organs with lowest similarity to the CAM (12% each). PECAM1, the gene encoding for the pan-endothelial marker CD31, was found in all organs, except liver, pancreas and kidney. PTPRF (protein tyrosine phosphatase, receptor type, F) also seems to have broad endothelial expression, since it is expressed in placenta, thyroid, lung, liver and brain (Supplementary figure S3 A).

**Table 5:** CAM development genes overexpressed in human organs.

Organ	Enriched unique transcripts	Shared with unique transcripts from CAM	%	Fold over Mean	Shared with unique EC-specific transcripts from CAM	%	Fold over Mean
**Placenta**	**1250**	**118**	**9.44**	**1.63**	**33**	**28**	**1.54**
**Lung**	**1702**	**137**	**8.05**	**1.39**	**28**	**20**	**1.13**
**Thyroid**	**1443**	**107**	**7.42**	**1.28**	**28**	**26**	**1.44**
Kidney	906	50	5.52	0.95	6	12	0.66
Pancreas	943	46	4.88	0.84	8	17	0.96
Blood	1862	83	4.46	0.77	14	17	0.93
Brain	1364	60	4.40	0.76	9	15	0.83
Liver	1725	70	4.06	0.70	11	16	0.87
Skin	889	34	3.82	0.66	4	12	0.65
**Mean**			**5.78**			**18**	

Comparison of human ortholog genes regulated during CAM development with genes overexpressed in indicated tissues. The highest percentage of common genes is found in organs functionally related to the CAM such as placenta and lung, but also in the highly vascularized thyroid. When only EC-enriched CAM genes were compared to the genes common between CAM and indicated organs, up to 28% of the genes were in common (for a list of these genes, see Supplementary figure S3).

CAM/Placenta and CAM/Lung-enriched genes showed a high degree of overlap with 63 shared genes (Supplementary figure S3 B). This set of genes expressed in organs with oxygen-delivering function contains critical factors such as CYR61, EPAS1, HES1, HEY1, ID3, KLF2, SOX7, RAMP2 and TFEB, which are known to play pivotal roles in vascular remodeling and morphogenesis. More than half (57%) of the genes shared between CAM and placenta and lung were also enriched in the thyroid (Supplementary figure S3 B). Interestingly, this set of genes also contained the previously described endothelium-enriched gene GPR116 (G protein-coupled receptor 116) [11] and C8orf4 (Thyroid cancer protein 1), which may play a role in thyroid cancer progression [34].

### Overexpression of EC-specific genes in human malignancies

**Figure 4:**
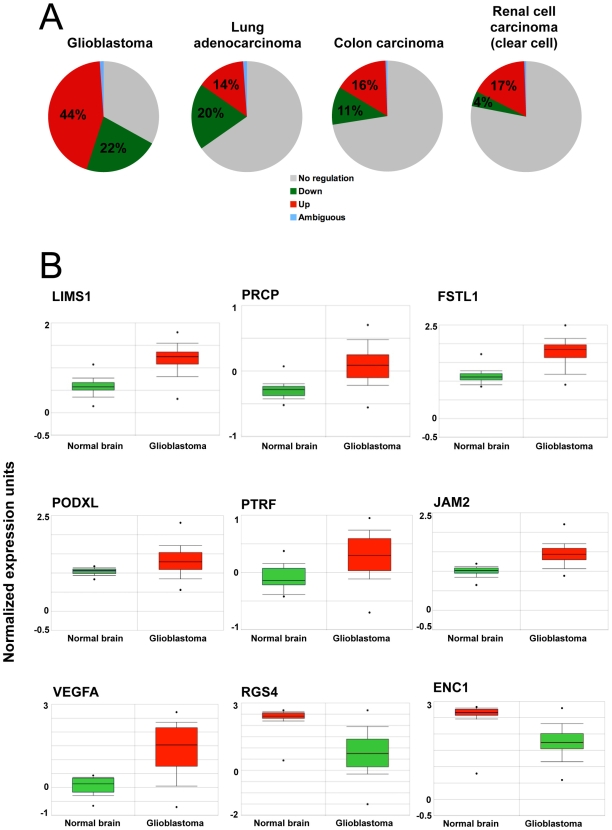
Overexpression of EC-enriched CAM genes in human cancers. A) Expression values of all genes with >2-fold EC enrichment were retrieved for glioblastoma, lung adenocarcinoma, colon carcinoma and clear cell renal carcinoma samples using Oncomine. 66% of these genes were found deregulated (over- and underexpression) in glioblastoma, 34% in lung adenocarcinoma, 27% in colon carcinoma and 21% in renal cancer, compared to normal non-malignant tissue. B) Detailed expression profiles for selected genes in glioblastoma. Red = overexpression, green = under expression; *P*<.0001 for all.

Genes with distinct functions in physiological angiogenesis are often deregulated in pathologies such as cancer and may play pivotal roles, thereby representing new therapeutic targets [35]. Using the Oncomine database [36], we determined the expression status of EC-enriched genes regulated during CAM development in four different cancer types which depend heavily on angiogenesis and which are to a certain degree sensitive to anti-angiogenic therapy. A large number of these genes were deregulated, up to 66% in glioblastoma, 34% in lung adenocarcinoma, 27% in colon carcinoma and 21% in renal cell carcinoma (Figure 4 A). Importantly, PRCP, PTRF, LIMS1 and FSTL1 were expressed in the developing murine brain vasculature (see Figure 3 M, O, P, Q) and are also overexpressed in glioblastoma (Figure 4 B), suggesting a role in tumor angiogenesis. As expected, VEGF was strongly overexpressed in glioblastoma samples, whereas RGS4 (regulator of G-protein signaling 4) [37], a negative regulator of VEGF-signaling, was downregulated.

### Effects of PARVB knock down in human EC cells

**Figure 5:**
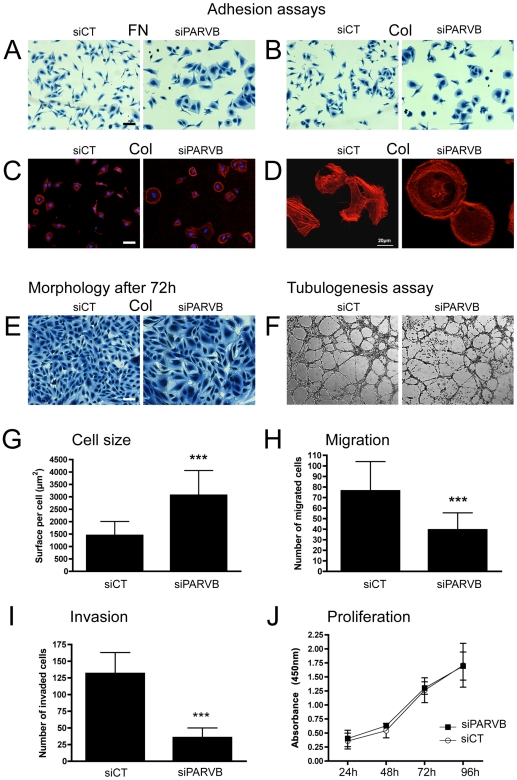
PARVB knock down in hCEMC/D3 ECs. A–E, G) Morphological changes during cell adhesion in siPARVB transfected cells. Increased cell size in the siPARVB treated cells can be observed as early as 3h after plating on fibronectin (FN; A) or collagen (COL; B), and differs significantly from control cells; G). C, D) Staining of the actin cytoskleton with rhodamine-phalloidin (20×). D) At higher magnification (D), note cytoskleton rearrangement with circular orientation and concentration of actin bundles at the cell borders. E) Altered EC morphology persists over time, up to 72h. H, I). Quantitative analysis shows that PARVB knock down leads to approximately two-fold decrease in cell migration compared to control (*P*<.0001) and 3.7-fold decrease in invasion through matrigel. J) Cell proliferation is not affected by PARVB knock down. F) Tubulogenesis is impaired by PARVB knock down. Cells stay isolated and tube-like structures are poorly organized (magnification 4×; all bars: 50µm).

We then explored functional implication of one of the CAM genes, PARVB, in the angiogenic process. We first determined efficacy of the PARVB siRNA in hCMEC/D3 endothelial cells. Target transcripts were potently reduced by more than 90% over a period up to 96h. transfected with PARVB siRNA showed profound changes in morphology as early as 3h after plating on collagen or fibronectin (Figure 5A, B). Cells transfected with non targeting siRNA (siCT) showed no morphological difference compared to untreated cells (data not shown). siPARVB cells appeared rounder and more spread-out compared to control cells. The culture dish area covered by a same number of cells was 2-fold for siPARVB (P<.0001) (Figure 5G). This phenomenon was observed after three hours, but not restricted to the initial adhesion, as siPARVB cells stayed larger over time, up to 72h (Figure 5E). The round phenotype of siPARVB cells is accompanied by a rearrangement of the actin cytoskleton as evidenced by rhodamine-phalloidin staining (Figure 5C, D). Actin fibers concentrate at the cell border and are orientated in a circular manner at the periphery.

We then challenged siPARVB cells in several functional tests to verify if the morphological changes affect cell motility. As seen in Figure 5H, short-term (3h) EC migration through transwell filters in response to serum was significantly reduced compared to controls (approx. 2-fold reduction). In an invasion assay, where cells have to migrate through Matrigel, a 3.7-fold reduction of siPARVB cells was evidenced (P<.0001). On the other hand, PARVB knock down had no significant effect on cell proliferation at any time point tested (Figure 5J). Endothelial cells dispersed in matrigel assemble into tube-like structures if stimulated by serum or growth factors (tubulogenesis assay). siPARVB cells had reduced ability to form tubes, with many single cells scattered in the gel which have not made contacts with other cells. Tubes themselves appeared thinner than controls (Figure 5 F).

## Discussion

In this study, we took advantage of the chick CAM as a unique vascular organ accessible for gene expression studies without any experimental manipulation of the tissue prior to mRNA isolation to identify new potential regulators of angiogenesis. Most cellular components of the CAM are either blood vessels and capillaries or epithelial cells in direct contact to capillaries. This makes the CAM an attractive model tissue to investigate gene regulation and expression throughout vascular development *in vivo*. We used cluster analysis, human ortholog screening, endothelial cell EST-to-gene assignments [32] and comparison with genes overexpressed in human tissues and tumors to focus on genes of possible relevance to human pathologies.

### Key modulators of angiogenesis are regulated during CAM maturation

It has been shown previously, that EC proliferation in the CAM peaks around day 10, then diminishes strongly [19], [20]. This active state of the endothelium is reflected by our study, with nearly 20-times more genes upregulated from E7 to E10, followed by a decrease in transcriptional activity after E10. Moreover, from E10 to E14, gene ontology analysis using DAVID [38], [39] found significant enrichment of mitosis regulators within the downregulated genes. On the other hand, from E7 to E10, genes were enriched that control blood vessel growth and morphogenesis, including BMP4, ACVR1, SMO and HOXA3. Interestingly, it has been suggested recently that HOXA3 expression provides positional cues during the development of the vasculature [40]. This study also validated endothelial HOXA3 protein expression, as predicted by our *in silico* analysis.

In vertebrates, vascular development is controlled by the hypoxia-inducible transcription factor EPAS1 [41], [42]. EPAS1 was significantly regulated during CAM development, reflecting the proliferation state of CAM ECs (e.g. upregulation from E7 to E10, followed by downregulation) and was strongly expressed in ECs, suggesting an active role of EPAS1 for CAM vascular development. Another gene selectively expressed in larger CAM vessels and whose regulation parallels EPAS1 from E10 to E14 is CYR61, which might have a role for vascular development in the CAM. A critical role in blood vessel development for this molecule has been demonstrated in CYR61 null mouse embryos, which are not viable because of severe vascular defects in the placenta [43].

Despite the fact that VEGF regulation did not reach significance in the SAM analysis, standard Affymetrix fold-change comparison showed a decrease of VEGF transcript levels by 50% from E10 to E14 (data not shown). This correlates well with the decrease in EC proliferation during this period.

FIGF, the gene for vascular endothelial growth factor D [44], exhibited a bi-phasic regulation pattern, with peaks at E7 and at E14. VEGF-D is strongly expressed in the mouse lung, promotes lymph- and hemangiogenesis, and is required for normal blood vessel formation in the zebrafish [45], [46]. It is possible that the first peak of FIGF regulation reflects initiation of lymphangiogenesis from E5 on. Noteworthy, from E5 to E7, FIGF is coregulated with its receptor FLT4. Downregulation of FIGF from E7 to E10 and then again upregulation to E14 - which is inverse to angiogenesis stimulation - suggests that VEGF-D mainly acts on the lymphatic system in the CAM or that its biological activity is controlled at the post-transcriptional level. It is known that processing of VEGF-D through cleavage occurs in the embryonic mouse lung, a step that greatly enhances its affinity for its receptors KDR and FLT4 [47]. Noteworthy, COL4A2, the precursor for the angiogenesis inhibitor canstatin [25], had the same regulation pattern as FIGF, and both genes are amongst the transcripts, which exhibit the highest fold-changes during CAM development. Co-expression of angiogenesis inhibitors and stimulators fits well in the concept of an angiogenic balance, where the effects of pro-angiogenic molecules are restricted by inhibitors [48].

Other critical genes, which control vascular morphogenesis, show significant regulation in our model. SDC2 (syndecan 2) knock-down in the zebrafish leads to defects in sprouting angiogenesis [49]. SDC2 is upregulated until E10 during CAM development, covering thus the whole period when sprouting angiogenesis occurs. UNC5B, also upregulated during the most active growth phase of the CAM, is expressed in ECs and its interaction with its ligand netrin-1 (encoded by NTN1) is essential for correct branching of the vasculature in mice and zebrafish [50].

### Novel genes with a functional role during vascular development

During the preparation of this manuscript, several other genes regulated between different stages of CAM maturation have been proven essential for normal vascular development in other species. In the early phase of the CAM, RSPO3 [51], [52] decreases 0.12-fold from E5 to E7, suggesting an important role of this factor at the early stages of the development of the CAM, even before E5, during the growth of the allantoic vesicle. Knockout of Rspo3 causes defects in the remodeling and establishment of placental and yolk sac vasculature in mice, leading to embryonic death around E10 [51], [52]. Speculative at this point, decreasing levels of RSP03 may reflect the switching from sprouting to intussusceptive angiogenesis in the developing CAM vasculature [21].

Krüppel-like transcription factors play important roles during development of the vascular system. KLF2 was upregulated between E5 and E7 of development, a period of active vascular expansion and recruitment of pericytes. KLF2 is not required for the development of a vascular plexus, but a failure in smooth muscle cell migration is observed in KLF null embryos, causing vascular defects, which lead to bleeding and lethality [18]. Interestingly, another Krüppel-like factor, KLF5 is also required for VSMCs recruitment during development and intima regeneration [53], [54].

RAMP2 was found expressed in ECs of the CAM. It is a co-receptor for adrenomedullin and is essential for developmental angiogenesis and lymphangiogenesis in mice [26], [27]. Strong and specific expression of RAMP2 in the CAM capillaries as well as larger vessels suggests a functional role of this molecule in CAM development.

SOX7 is expressed in the developing Xenopus vasculature [55] and ECs in the CAM and recent functional studies in zebrafish revealed a redundant, yet essential role for SOX7 together with the related SOX18 in vascular development [56], [57], [58].

Genes of a same functional network may be co-regulated during vascular maturation. Myc null mice die around E10.5 due to major defects in the developing vasculature [59]. SNAI2 and TWIST1, two recently discovered downstream mediators of MYC activity on vascular development in *Xenopus laevis* [31], are downregulated together with MYC after E10 of CAM development, underlining the importance of this regulatory circuit. Interestingly, SNAI2 is strongly expressed in pericytes during chick development [60].

### CAM genes in human tissues

The CAM is the central oxygen-exchanging organ for the developing chick embryo; its function thus shares features with organs like the placenta and the lung. We therefore compared genes regulated in the CAM to sets of genes overexpressed in human tissues, including placenta and lung [61]. A set of 63 human orthologs is conserved between lung, placenta and the CAM. This set of genes contained transcription factors which control lung and/or placenta morphogenesis and vascularization such as EPAS1 [41], HEY1 [62], and other critical genes such as CYR61 and RAMP2 which also may play roles in placenta pathology [63], [64]. Interestingly, 34% of the genes shared between the CAM and the thyroid, are also found in the CAM/placenta/lung expressed genes. Even though the thyroid is functionally not related to placenta or lung, it is a highly vascularized organ and therefore may contain more EC transcripts than other tissues.

Two other transcriptomic studies of ECs isolated from mouse lungs and other tissues [5], [11] have identified numerous genes enriched in the endothelial compartment. When intersected with EC-enriched genes of the CAM transcriptome (Supplementary table S3), only very few genes are found in common (2.8% for each study). These genes are HEY1, ITGA6, PECAM1, TNFSF10 and TSPAN7 (from the Favre study [5]) and EPAS1, GPR116, PTPRM, SOX7 and TNFSF10 (from the Wallgard study [11]). When the Favre and Wallgard studies are compared, 13.7% of the reported genes are common (data not shown).

The reason for this low overlap might lie in the different methods used to isolate mRNA, and the different filters that we specifically applied. We introduced a dynamic parameter, e.g. regulation between two time points, which obviously eliminates genes that are constantly expressed by the endothelium. Also, we included only genes with human orthologs, which limits comparison with mouse genes. Last, we restricted our analysis to genes enriched in endothelial ESTs libraries. Various, complementary approaches are still needed to identify possible future candidates critical for vascular development.

### New potential regulators

The fact that a large number of essential modulators of angiogenesis participate in CAM vascular development suggests that genes previously not associated with blood vessel formation might be present in the gene signatures we have described. For example, class 3 semaphorins, such as SEMA3A and –F, which are secreted neuronal guidance proteins, are also negatively regulating blood vessel branching [65]. SEMA3C, which is associated with CAM vascular maturation, has not been investigated *in vivo*, although it stimulates EC proliferation and survival *in vitro* [66].

PTRF displays a high EC-enrichment (>12-fold) and is expressed in brain capillaries. It encodes a protein found in caveolae and is essential for their function [67]. Caveolae are central structural components of ECs and control cell behavior and signaling [68]. Furthermore, PTRF was found overexpressed in glioblastoma.

PARVB, encodes beta-parvin, which negatively regulates integrin-linked kinase (ILK) activity in breast cancer cells [69]. Integrin-linked kinase activity in the endothelium or in tumor cells is essential for physiological and pathological angiogenesis [70], [71], [72]. Therefore, PARVB expression in ECs suggests the existence of a negative feedback loop on integrin-linked kinase activity, which may be important during vascular development. Since no direct data exist about the role of PARVB in angiogenesis, we investigated the effects of PARVB knock down in human immortalized capillary endothelial cells [73]. PARVB depletion alters EC adhesion, migration, invasion and tubulogenesis, suggesting that Beta-parvin may play a role in modulating angiogenesis.

It seems that Beta-parvin activity needs to be tightly controlled, since overexpression of a ILK- phosphorylated COOH-terminal fragment of Beta-parvin, blocks cell spreading of CHO cells at the initial stage [74]. Another domain of Beta-parvin, which interacts with alpha-actinin, is also implicated in cell adhesion and loss/gain-of-function experiments have shown deleterious effects on cell adhesion [75]. Whether effects of PARVB knock down in ECs is a direct or indirect effect mediated by deregulated integrin-linked kinase activity remains speculative at this point. Interestingly, both PARVA and PARVB are down-regulated during the later phase of the CAM, when the endothelium becomes quiescent, suggesting an implication of these two genes earlier in CAM angiogenesis.

Another gene with high EC-specificity is PODXL (podocalyxin-like). However, PODXL, highly homologous to CD34, seems to have redundant roles in vascular development, since Pdxl (−/−) mice show no vascular abnormalities [76]. PODXL is nonetheless significantly overexpressed in glioblastoma, suggesting a possible implication in tumor angiogenesis.

Some attractive gene candidates identified in this study are expressed elsewhere than in ECs. We found transcripts for dystroglycan (DAG1) in the chorionic epithelium, which is in close contact with the capillary network. Dystroglycan may modulate angiogenesis via interaction with the alpha chain of laminin-1, the gene product of LAMA1, which is downregulated with DAG1 from E10 to E14 [33], [77]). Based on our bioinformatic analysis, several genes preferentially expressed in stromal cells may play critical roles in CAM vascular maturation (see Supplementary table S4). An interesting candidate is MDK encoding for Midkine, a heparin-binding growth factor. Epithelial-expressed Midkine controls lung vascular morphogenesis via upregulation of genes critical for vascular smooth muscle cell function in a HIF-1alpha-dependent manner [13]. Stromal cells might also restrict vascular growth by producing precursor proteins for angiogenesis inhibitors. We found genes encoding calreticulin (CALR) as well as collagen 4A2 (COL4A2) enriched in the stromal compartement of the CAM. Vasostatin, [78] a fragment of calreticulin and canstatin, derived from the alpha2 chain of type IV collagen [25] might negatively control vascular growth in the developing CAM and perhaps restrict excessive angiogenesis. Interestingly, COL4A2 is the most down regulated gene during CAM angiogenesis from E7 to E10, when most genes are upregulated.

Importantly, some of the CAM development genes over expressed in ECs might be markers of poor prognosis in human cancer. We found FSTL1 transcripts in brain capillaries and over expressed in glioblastoma samples. Importantly, a recent study confirmed association of FSLT1 over expression with poor prognosis in glioblastoma patients [79]. FSTL1 is down regulated after E10 in the CAM, at a time point when angiogenesis in the CAM declines. FSTL1 might exert pro-angiogenic effects (survival, migration) during development and tumor angiogenesis in a similar manner as has been shown *in vitro* and in ischemic tissue [80]. The exact role of other vascular genes such as PRCP, LIMS1 and ENC1 in glioblastoma is not clear yet, but given their expression pattern, an implication in tumor angiogenesis is possible and merits further investigation.

Other genes, which are significantly regulated during CAM development, play also central roles in human diseases with vascular anomalies. TIMP3 mutations cause Sorby's fundus dystrophy, a disease, where choroidal vessel integrity is affected; SOX18 inactivation leads to lymphedema-distichiasis syndrome, with clinical signs of edema and abnormal morphology of capillaries and arterioles and FLT4 mutations cause Nonne-Milroy lymphedema (for review see [81]).

### Conclusions

Taken together, the CAM transcriptome constitutes the first detailed *in vivo* analysis of a vascular tissue during its maturation. Our data show that the development of the vasculature in the CAM of the chick embryo involves regulation of critical genes, which have been reported to control vascular morphogenesis in far more complex tissues, such as placenta and lung and which also show deregulation in various human cancers, suggesting that these new genes are involved in human pathologies in which vascular growth and remodeling plays a central role (e.g. tumor angiogenesis).

## Materials and Methods

### Analysis of CAM development by biomicroscopy and immunhistochemistry

The evolution of the CAM vascular network was visualized from E5 to E14 by standard biomicroscopy at 10–63× magnifications. Immunhistochemistry of CAM whole mounts was performed as described previously [82]. Lymphatic endothelial cells were identified using a rabbit anti-Prox-1 antibody (ab11941, 1∶500, Abcam, Paris, France) and pericytes were visualized with a rabbit anti-desmin (clone D33, 1∶500, Dako, Treppes Cedex, France). Corresponding secondary antibodies were coupled to Alexa Fluor 546 (1∶2000, Invitrogen, Cergy Pontoise Cedex, France).

### Isolation of CAM tissue for gene profiling

The developmental stage of the embryos was determined after isolation of the CAM according to Hamburger & Hamilton (HH) [83], and only CAMs from typical embryos were used. CAMs were isolated from embryos at developmental day E5 (HH26), E7 (HH30), E10 (HH>35) and E14 (HH40), and directly snap-frozen in liquid nitrogen mRNA was isolated using RNeasy minikit (Qiagen, Courtaboeuf cedex, France) and hybridized to Affymetrix chicken GeneChips using the manufacturer's standard protocol (Affymetrix UK Ltd, High Wycombe, UK).

### Affymetrix chicken GeneChips: comparison set-up and statistical analysis

The chicken GeneChip covers 32773 transcripts corresponding to >28000 chicken genes, has a probe set oligonucleotide length of 25 and a detection sensitivity of 1∶100000 (http://www.affymetrix.com). Data were analyzed with the GCOS 1.2 software (Affymetrix), using the default analysis settings and global scaling as first normalization method, with a trimmed mean target intensity value (TGT) of each array arbitrarily set to 100.

Three individual CAMs of a same developmental stage were compared to three CAMs from a more advanced stage. The raw data set was filtered prior to further statistical analysis using the following criteria: each probe set had to be labeled as “present” (*P*-value<.04, Wilcoxon rank sum test) in all three embryos on at least one developmental day. This set of 17778 probe sets was subjected to Significance Analysis of Microarrays (SAM) analysis [84] (http://www-stat.stanford.edu/~tibs/SAM/, which utilizes a Wilcoxon-test statistic and sample-label permutation to evaluate statistical significance between sample groups. SAM provides mean fold change values (FC) (mean fold-change >2) and a false discovery rate (FDR) confidence percentage based on data permutation (n = 200). The False Discovery Rate (FDR), an estimate of the fraction of selective genes, was kept below 5% in all statistical analyses (the graphical representation of the SAM analysis is shown in Supplementary figure S1). Annotation of genes was performed using NetAffx (http://www.affymetrix.com; March 2009). The 12 microarray data files have been submitted to the US National Center for Biotechnology Information, Gene Expression Omnibus (GEO), and will be released upon publication (approved accession number: GSE11636; sample numbers: GSM294982, GSM294983, GSM294984, GSM294985, GSM294986, GSM294987, GSM294988, GSM294989, GSM294990, GSM294991, GSM294992, GSM294993).

### Cluster analysis

To visualize expression values of genes identified as being significantly regulated by the SAM analysis, we performed a Cluster analysis between E5 and E7, E7 and E10 and E10 and E14 (Cluster 3 for Mac OSX). Original hybridization signal data were log transformed and median centered prior clustering (hierarchical cluster for genes, correlation uncentered with average linkage). The cluster was visualized (Java TreeView, Version 1.0.13) and genes known to control vascular morphogenesis were searched. The aim was to evidence co-expression of genes implicated in the same functional network or process or which physically interact. Neighboring genes were displayed by selecting the node close to the gene of interest (in red), co-expressed angiogenesis genes were labeled in blue and genes with EC-enrichment (from Supplementary table S3) indicated by an arrow.

### Gene Ontology analysis

To get insight into the biological processes associated with the regulated genes between the different periods, Affymetrix IDs were submitted to the DAVID (Database for Annotation, Visualization and Integrated Discovery) analysis tool [38], [39]. We submitted all probes upregulated from E5 to E7, from E7 to E10 (maximal growth phase) and all probes downregulated from E10 to 14 (period in which the CAM vasculature gets quiescent) and performed functional annotation clustering, searching for biological processes, which were enriched in our gene sets compared to the rest of the chicken genome. All results are ranked based on a FDR-based q-value and *P*-value (from a modified Fisher Exact test) and processes (Gene Ontology “Biological Process” category) with the highest enrichment were selected (for more information about DAVID, please visit http://david.abcc.ncifcrf.gov/home.jsp).

### Semi-quantitative real-time PCR

Total RNA was purified from pools of 10 CAMs per developmental day using RNeasy columns (Qiagen, Courtaboeuf Cedex, France). RNA was reversed-transcribed with SuperScript II RNase H- Reverse Transcriptase (Invitrogen, Cergy Pontoise Cedex, France) by using oligo (dT)15 priming. Chicken-specific primers for qPCR were designed and evaluated for amplification efficiency. Primer sequences were: HNRPH1, 5′- GCTGTGTCTGCCACGAGTTA -3′, 5′- GCTTTCGGCTGAGAGACAAT-3′, EPAS1, 5′- AATCCACCTGTGGCAGTCCT-3′, 5′- AAGACCCCAGCAGACGACTC-3′ and CYR61, 5′- AGCTCTTCCTCTCCCGTTCA-3′, 5′-ACAAGTGCCCACCTCAGGAA-3′. Real-time PCR was carried out in a MX3000P thermocycler (Stratagene) by using SYBR Green dye (ABgene, Courtaboeuf Cedex; France). Normalization and quantification were calculated according to the formula 2^ΔΔCt^. PCRs were performed independently 3 times.

### Human-chick ortholog identification : analysis of the BLAST pipeline results

From the SAM analyses, there were 1252 (1176 non-redundant) differentially expressed probes from the different conditions. The bioinformatic goal was to identify the human ortholog of chicken gene represented by the Affymetrix probes. A graph based, nearest neighbour approach, Reciprocal Best Hit (RBH) [85], was chosen in preference to multiple sequence alignments and phylogenetic tree analyses, as performing those was not amenable to high-throughput of the 1252 probes. Coupled to this was the fact that a recent performance assessment of different ortholog prediction methods found RBH performed well in comparison with other methods [86]. An RBH approach uses BLAST to compare a query sequence against another genome [85], [87]. If the best gene from the other genome best matches the original query sequence it is termed a Reciprocal Best Hit. An RBH analysis was carried out in three ways and the results combined to give the maximum number of successful RBH human ortholog assignments.

Method 1: Affymetrix annotation data file, dated 3^rd^ March 2009 (Chicken.na28.annot.csv), was downloaded from Affymetrix technical support website: http://www.affymetrix.com/support/technical/annotationfilesmain.affx. 867 of the 1176 non-redundant probes were annotated with a chicken protein accession by Affymetrix. The chicken protein annotated in the Affymetrix file was used as a query to search the human Refseq database of proteins to assign orthologs by RBH. 745 of the 1176 non-redundant probes were successfully assigned human orthologs.

Method 2: In this method, the accession nucleotide accessions in the Chicken.na28.annot.csv file were collected and sequences used to find the best chicken Refseq nucleotide. If the alignment was of sufficient quality, >90% sequence identity and > = 100bp alignment length, then the protein sequence for the Refseq nucleotide was used in a RBH analysis versus human proteins. 765 of the 1176 non-redundant probes were successfully assigned a human ortholog using this method.

Method 3: A high-throughput method of assigning orthologs through a Conditional Stepped Reciprocal Best approach (CSRBH) (Herbert et al., manuscript under review) was used to assign all probes a human ortholog. The nucleotide sequence was first translated in six frames and BLAST searched against the chicken and human Refseq protein databases. Whichever species gave the most similar protein match, this protein used in a RBH analysis to assign the ortholog. Therefore, if a chicken protein was the most significant match to the nucleotide sequence, then that chicken protein was used in a RBH analysis to assign a human ortholog. Alternatively, if there was no chicken protein more similar than a human protein, then the human protein was used in the RBH analysis. The number of successful RBH human orthologs assignments using this method was 719.

Combining results: By combining the three RBH methods of ortholog assignments, 912 of the 1176 non-redundant probes were successfully assigned human orthologs. Applying this to the original list of 1252 redundant probes, 946 of them were successfully assigned a human ortholog.

### *In silico* EC and Non-EC gene enrichment prediction

To assign gene regulation in the CAM to specific cell types (EC vs. Non-EC: stromal cells), we relied on a recent bioinformatic approach. Preferentially expressed genes in cDNA libraries isolated from endothelial cells (EC-ESTs, n = 31114 ESTs) vs. non-endothelial cells (Non-EC-EST, n = 136622 ESTs) were identified as described (Herbert et al. 2008 [32]). In their work, a False Discovery Rate q-value [88], [89s] of <0.01 was used to define endothelial differentially expressed genes. Although the authors found highly endothelial specific genes applying this q-value, in this work it was considered too stringent as endothelial genes expressed at a low level could be missed because of low coverage sequencing of cDNA libraries. Therefore, a less stringent EC-enrichment fold-ratio method was used, which was calculated by dividing the number of transcripts from the EC pool by the number of transcripts from the non-EC pool (per million transcripts). A two-fold upregulation threshold in endothelial cells was applied and the q-value reported. Non-EC identity was assigned to genes using the same criteria.

### Bioinformatic tools

The bioinformatic analyses performed in this work can be done locally on user data and can be downloaded from: http://www.cbrg.ox.ac.uk/~jherbert/

### Cloning of chick genes and *in situ* hybridization

Sense and antisense riboprobes (800–2000 bp 3′UTR) were prepared from cDNA of E7 CAM. Briefly, for SOX7, CYR61, RAMP2 and DAG1 PCR products were amplified with indicated chicken-specific primers and cloned into TOPO TA Cloning Kit (with pCR 2.1-TOPO, Invitrogen). SOX7 (GgaAffx.20877.1.S1_at, XM_001234627; 997nt), F: TGCTTAGGTAAAGGATTTCG, R: GAAAATCATAGCCACGCTAC. CYR61 (GgaAffx.20877.1.S1_at XM_001234627; 1375nt), F: GAGACCATGCGAATACAACT, R: TTCCAGTATTACAGGGGTTG. RAMP2 (Gga.1941.1.S1_at NM_204099; 831nt). F: CCCAGACAGGATTAAAGAGA, R: TAAACCTTTACTGCCCGATG. DAG1 (GgaAffx.20323.1.S1_at NM_001097540; 1992 nt), F: ATGACTGTTGGATGTGTCC, R: GGTGTTGTTGGTCCATTC. Plasmid carrying EPAS1 cDNA was kindly provided by J. Favier (INSERM U977, France) [90]. *In situ* hybridization was carried out essentially as described [91]. The genepaint database ([92]; http://www.genepaint.org) that provides digital *in situ* hybridization images of sagittal sections of E14.5 mouse embryos was queried for all genes indicated in Supplementary table S3. Positive signal in capillaries of different organs was evidenced by comparing to the expression pattern of known highly-specific endothelial-specific genes CDH5 (VE-cadherin) or PECAM1 (CD31).

### Comparison of CAM genes with other organs

Genes enriched in indicated human organs were retrieved from the GNF SymAtlas v1.2.4, (http://symatlas.gnf.org/SymAtlas/ [61]). Transcripts with a three-fold enrichment over median expression in 79 other tissues (MAS5 normalization) in lung (n = 1804), placenta (n = 1320) whole brain (n = 1462), whole blood (n = 1968), kidney (n = 1121), pancreas (n = 1154), liver (n = 1875), skin (n = 1245) and thyroid (n = 1483) were selected. These gene lists were compared to high quality orthologs regulated significantly during CAM development (n = 946 probe sets) using a web-based gene comparison tool (http://elegans.uky.edu/MA/progs/Compare.html). Unique genes shared between the developing CAM and other organs are expressed as percentage of genes enriched in the indicated organ.

### Expression of EC-enriched genes in human cancers

Genes playing key roles during developmental angiogenesis are often deregulated during pathological angiogenesis in solid tumors [35]. To establish a link between CAM development genes and tumor angiogenesis, we retrieved expression levels of all genes with an EC-enrichement >2 (n = 178) in four solid tumors (glioblastoma, lung adenocarcinoma, colon carcinoma and renal clear cell carcinoma) using the Oncomine database ([93]; http://www.oncomine.org). Oncomine centralizes expression profiles of over 3762 microarray experiments covering a broad panel of human cancers and normal tissues and can be used to link expression of novel genes with different grades of tumors, tumor sub-types and, as has been recently shown for CD200 [94]. Expression levels for selected genes were demonstrated for glioblastoma, one of the most highly vascularized tumors. VEGF served as positive control [95]. Expression was considered different to normal tissue, when was *P*<.0001). Glioblastoma data originated from the study of Sun et al. [96].

### Functional analyses of PARVB knock down in human endothelial cells

#### *EC cell culture*

hCMEC/D3 cells [73], (a kind gift of P.O. Couraud, Institut Cochin, Paris) were maintained as described [73]. Briefly, cells were plated onto type I collagen coated dishes (rat tail collagen type I, 100µg/mL, BD Biosciences) in endothelial basal medium (EBM2) (Lonza, Levallois-Perret cedex, France) containing FBS and growth supplements (EGM2-MV) as recommended by the manufacturers. The cultures were maintained at 37°C in 5% CO2.

### siRNAs and transfection

ON-TARGETplus siRNA against human PARVB (siPARVB) (target sequence: G.G.A.A.G.A.A.C.C.U.G.G.U.G.G.C.C.A.U) and ON-TARGETplus non-targeting siRNA (siCT) were designed by Dharmacon (Thermofisher scientific, Courtaboeuf cedex, France) and transfected into cells using Lipofectamine RNAiMAX (Invitrogen, Cergy Pontoise cedex, France) according to the manufacturers indications. Efficacy of PARVB knock down was tested by semi-quantitative real-time PCR using the following primers for PARVB: F: ATGGCGTGTACCTGGTTCTG; R: AAGGCGAAGGACACATTGTGG.

### EC migration, invasion, adhesion and proliferation

Migration and invasion of siCT and siPARVB-transfected cells was evaluated using transwell chambers with a 8-µm pore size polycarbonate membrane (Falcon, BD Bioscience, Le Pont de Claix cedex, France), coated with type I collagen (100µg/ml) or matrigel (20µg/100µl) respectively. The lower chambers were filled with endothelial basal medium with or without serum (0.5% and 0.1% for migration and invasion, respectively). 5×10^4^ cells were seeded for migration assays and 1×10^5^ cells for invasion assays and incubated at 37°C for 3h (migration), and 24h for invasion. Then, the non-migrated cells on the upper side of filters were cleared with cotton swabs, and the migrated cells were fixed with an acetic acid/MetOH solution (10%/30%) and stained with Coomassie blue. The number of migrated cells were counted by light microscopy (4 fields per inserts). Experiments were repeated three to four times, with very reproducible results. All data are pooled and analyzed using unpaired t-tests.

For adhesion assay and cell morphology studies, 3×10^3^ cells were plated onto collagen-coated 96-wells plate, in EBM2 medium plus BSA 1%. After 3 to 72h of adhesion, cells were rinsed three times with PBS, fixed with an acetic acid/MetOH solution (10%/30%) and stained with Coomassie blue. To quantify cell size at 3 hours of adhesion, total cell number was determined based on coomassie staining under light microscope, total area occupied by cells was determined by the imaging software (NIS-Elements AR 2.30 software; Nikon France), and mean surface per cell was calculated (area/number of cells). The experiment was repeated three times, and results pooled for statistical analysis (Mann-Whitneys U-test).

For actin cytoskeleton staining, 3,5.10^4^ hCMEC/D3 cells were plated onto collagen-coated coverslides, in EBM2 medium plus BSA 1%, and placed in 24 wells plate. Cell adhesion was allowed for 3 hours at 37°C and 5% CO2. After 3 PBS washes, cells were fixed with paraformaldehyde, permeabilized with PBS/Triton 1% and saturated with BSA 5%. Rhodamine-phalloidin (Molecular Probes) staining was performed to reveal actin cytoskeleton. Images were acquired using a confocal microscope (Leica SP5).

For proliferation assay, 3×10^3^ hCMEC/D3 cells were seeded in 96-well plates in complete medium. After transfection with siARN PARVB or siCT, cell proliferation was assessed by colorimetric WST-1 assays (Roche, Neuilly sur Seine Cedex, France) at indicated time points, according to manufacturers indications. The results are expressed as raw values corrected for background absorption. Five wells per day and condition were used and two independent experiments pooled. Statistical analysis was done using Mann-Whitneys U-test.

### Tubulogenesis assay

25×10^4^ hCMEC/D3 were transfected with siRNAs for 72h, mixed with matrigel on ice and then transferred into 24-well culture dishes (350µl per well). EBM2 medium containing 0.1% serum (hCMEC/D3) was then added and cells were incubated at 37°C. After 18–20h, photos were taken using a Leica light microscope. Ten wells per condition were analyzed, and one representative image is shown.

## Supporting Information

Figure S1 Graphical representation of the SAM analysis. Each SAM curve contains all the genes plotted by their observed scores and expected scores. The red, green, and black dots represent up regulated, down regulated, and insignificant genes respectively. The upper and lower 45° degree lines indicate the Δ threshold boundaries. Genes with Δ = 0 would fall on the 45° line through the origin. The number of significant genes, median number of false positives, and false discovery rate are indicated at the upper left corner of each plot sheet.
(1.16 MB TIF)

Figure S2 Functional annotation clustering using DAVID. To gain insight into biological processes enriched during CAM development, we submitted significantly regulated genes to Gene Ontology via DAVID. Biological processes, which were significantly enriched in the submitted set of genes compared to the rest of the genome, are indicated.
(1.49 MB TIF)

Figure S3 Comparison of CAM development genes and genes over expressed in human organs. A) Predicted EC-specific genes enriched in indicated organs. B) Intersection of over expressed genes shared between the CAM and the three indicated organs. This gene set contained numerous known key regulators of placenta and lung morphogenesis and vascularization.
(1.55 MB TIF)

Table S1 Full list of significantly regulated genes (>2-fold). The Excel file contains up- and down regulated genes for each developmental period compared: E5 vs. E7, E7 vs. E10 and E10 vs E14.
(0.23 MB XLS)

Table S2 Ortholog identification process. This table contains the ortholog screening process for all probes for which significant changes have been found using the SAM analysis.
(0.29 MB XLS)

Table S3 Full list of putative human orthologs of CAM genes with endothelial specificity (>2-fold enrichment). This table contains 205 probe sets (178 unique genes) with significant changes during indicated period of development. The 2-fold enrichment indicates two times more transcripts found in the EC-EST pool. 1: over expression in tumors; −1: under expression; 0: no regulation; AMB: ambiguous regulation (over and under expression found). Unique genes were identified using http://elegans.uky.edu/MA/progs/Compare.html.
(0.06 MB XLS)

Table S4 Full list of putative human orthologs of CAM genes with non-endothelial specificity (>2-fold enrichment). This table contains 354 probe sets (297 unique genes) with significant changes during indicated period of development. The 2-fold enrichment indicates two times more transcripts found in the Non-EC-EST pool. Unique genes were identified using http://elegans.uky.edu/MA/progs/Compare.html.
(0.08 MB XLS)
