# Correction: Correlating Global Gene Regulation to Angiogenesis in the Developing Chick Extra-Embryonic Vascular System

**DOI:** 10.1371/annotation/110bdc8c-b16e-4043-816c-a3f8105ca061

**Published:** 2010-03-23

**Authors:** Sophie Javerzat, Mélanie Franco, John Herbert, Natalia Platonova, Anne-Lise Peille, Véronique Pantesco, John De Vos, Said Assou, Roy Bicknell, Andreas Bikfalvi, Martin Hagedorn

A funding source was omitted from this article. Part of this work was financed by the ARC (Association pour la Recherche sur le Cancer), grant A08 / 5 / 1062 to MH.

